# A solution to the challenges of interdisciplinary aggregation and use of specimen-level trait data

**DOI:** 10.1016/j.isci.2022.105101

**Published:** 2022-09-13

**Authors:** Meghan A. Balk, John Deck, Kitty F. Emery, Ramona L. Walls, Dana Reuter, Raphael LaFrance, Joaquín Arroyo-Cabrales, Paul Barrett, Jessica Blois, Arianne Boileau, Laura Brenskelle, Nicole R. Cannarozzi, J. Alberto Cruz, Liliana M. Dávalos, Noé U. de la Sancha, Prasiddhi Gyawali, Maggie M. Hantak, Samantha Hopkins, Brooks Kohli, Jessica N. King, Michelle S. Koo, A. Michelle Lawing, Helena Machado, Samantha M. McCrane, Bryan McLean, Michèle E. Morgan, Suzanne Pilaar Birch, Denne Reed, Elizabeth J. Reitz, Neeka Sewnath, Nathan S. Upham, Amelia Villaseñor, Laurel Yohe, Edward B. Davis, Robert P. Guralnick

**Affiliations:** 1 National Ecology Observatory Network, Battelle, Boulder, CO 80301, USA; 2 Smithsonian Institution, National Museum of Natural History, Washington, DC 20560, USA; 3 Berkeley Natural History Museums, University of California, Berkeley, Berkeley, CA 94720, USA; 4 Biocode LLC, Junction City, OR 97448, USA; 5 Florida Museum of Natural History, University of Florida, Gainesville, FL 32611, USA; 6 Critical Path Institute, Tucson, AZ 85718, USA; 7 BIO5 Institute, University of Arizona, Tucson, AZ 85719, USA; 8 Department of Earth Sciences, University of Oregon, Eugene, OR 97403, USA; 9 Archaeozoology Lab, Instituto Nacional de Antropologia e Historia, 06060 Mexico City, CdMx, Mexico; 10 Department of Life and Environmental Sciences, University of California, Merced, Merced, CA 95343, USA; 11 Department of Archaeology, Simon Fraser University, Burnaby, BC V5A 1S6, Canada; 12 Department of Biology, University of Florida, Gainesville, FL 32611, USA; 13 Stony Brook University, Stony Brook, NY 11794, USA; 14 Department of Environmental Science and Studies, DePaul University, Chicago, IL 60614, USA; 15 Negaunee Integrative Research Center, The Field Museum of Natural History, Chicago, IL 60605, USA; 16 College of Science, University of Arizona, Tucson, AZ 85721, USA; 17 Museum of Natural and Cultural History, University of Oregon, Eugene, OR 97401, USA; 18 Department of Biology and Chemistry, Morehead State University, Morehead, KY 40351, USA; 19 Museum of Vertebrate Zoology, University of California, Berkeley, Berkeley, CA 94720, USA; 20 Department of Ecology and Conservation Biology, Texas A&M University, College Station, TX 77843, USA; 21 Department of Anthropology, University of Florida, Gainesville, FL 32611, USA; 22 Department of Biology, University of North Carolina, Greensboro, NC 27412, USA; 23 Peabody Museum of Archaeology and Ethnology, Harvard University, Cambridge, MA 02138, USA; 24 Department of Anthropology, University of Georgia, Athens, GA 30602, USA; 25 Department of Geography, University of Georgia, Athens, GA 30602, USA; 26 Department of Anthropology, University of Texas, Austin, Austin, TX 78712, USA; 27 Georgia Museum of Natural History, University of Georgia, Athens, GA 30602, USA; 28 School of Life Sciences, Arizona State University, Tempe, AZ 85281, USA; 29 Department of Anthropology, University of Arkansas, Fayetteville, AR 72701, USA; 30 Department of Bioinformatics and Genomics, University of North Carolina Charlotte, Charlotte, NC 28223, USA

**Keywords:** Ornithology, Animals, Systematics, Evolutionary history, Phylogenetics, Biological database, Paleobiology

## Abstract

Understanding variation of traits within and among species through time and across space is central to many questions in biology. Many resources assemble species-level trait data, but the data and metadata underlying those trait measurements are often not reported. Here, we introduce FuTRES (Functional Trait Resource for Environmental Studies; pronounced few-tress), an online datastore and community resource for individual-level trait reporting that utilizes a semantic framework. FuTRES already stores millions of trait measurements for paleobiological, zooarchaeological, and modern specimens, with a current focus on mammals. We compare dynamically derived extant mammal species' body size measurements in FuTRES with summary values from other compilations, highlighting potential issues with simply reporting a single mean estimate. We then show that individual-level data improve estimates of body mass—including uncertainty—for zooarchaeological specimens. FuTRES facilitates trait data integration and discoverability, accelerating new research agendas, especially scaling from intra- to interspecific trait variability.

## Highlights

• Functional Trait Resource for Environmental Studies (FuTRES; few-tress)
• Individual-level trait datastore for paleo-, zooarcheological, and modern specimens
• Millions of individual-level trait data records already available for mammals
• Semantic framework for enhanced interoperability, R package for access, and APIas

## Graphical abstract

**Figure:**
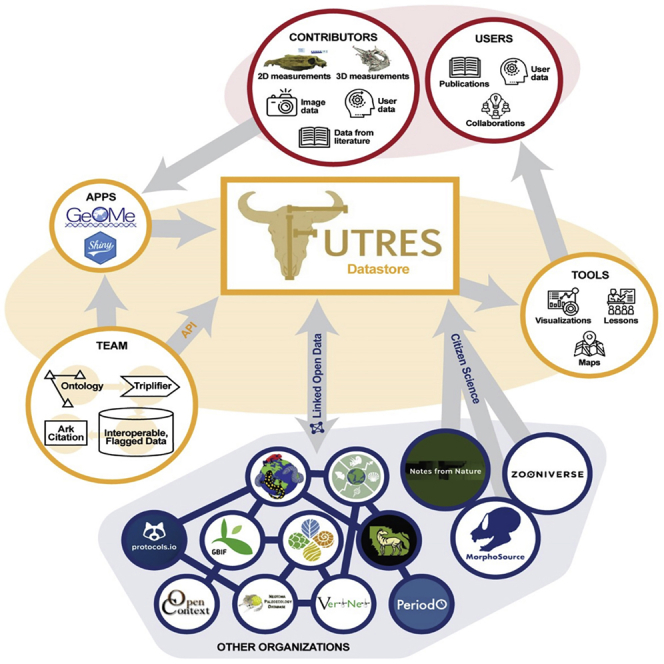

## Introduction

Traits are the measurable morphological, physiological, behavioral, and life-history characteristics of organisms that directly interact with the environment and thus determine how organisms respond to changing environmental conditions (Eronen et al., 2010; Polly et al., 2011; Guralnick et al., 2020; Saarinen et al., 2021). Trait-based approaches in ecology are vital as new theoretical and empirical efforts have led to novel insights about linkages between traits and niche overlap at the population and community levels (McGill et al., 2006; Violle et al., 2014; Read et al., 2018), as well as the importance of traits in structuring composition of assemblages (Ackerly and Cornwell, 2007; Holt et al., 2018). These approaches also have been crucial for asking and answering time-extended, macroevolutionary questions, such as relationships between rates of trait evolution and species diversification (Folk et al., 2019; Upham et al., 2020), patterns of functional diversity along gradients at varying scales (Cisneros et al., 2014; Dreiss et al., 2015; de la Sancha et al., 2020), adaptive and plastic responses of traits to past environmental change (Smith and Betancourt, 2006; Saarinen et al., 2021), and human modification of the environment (Tomé et al., 2019; Hill et al., 2008; Guthrie, 2003). Thus, trait-based approaches will continue to connect within and across disciplines, providing a common framework across not only ecology and evolution but also paleontology and environmental archaeology.

Given the centrality of traits in modern biology, it is unsurprising that many trait databases have recently emerged, typically (Smith et al., 2003; Jones et al., 2009; Gallagher et al., 2020), but not always (Kattge et al., 2011; Gonçalves et al., 2018), built by extracting information from existing literature. Focusing here on vertebrates, such compilations typically cover key life-history information such as number of offspring, body size, or even equations for estimating body size (Jones et al., 2009; Smith et al., 2003; Wilman et al., 2014; Damuth and MacFadden, 1990; Myhryold et al., 2015). While impactful in enabling macroscale research, these compilations usually only report species’ mean or maximum values (e.g., Jones et al., 2009; Smith et al., 2003; Wilman et al., 2014; Damuth and MacFadden, 1990), or value ranges (Myers et al., 2020). Emphasis on means or ranges fundamentally limits the utility of these trait databases for many biodiversity-based research studies. These summaries have been built *de facto* from measurements of traits of individuals, but neither the measurements of the traits themselves nor their provenance are typically maintained, except in unpublished original field and lab records. In particular, critical metadata about the specimens on which they were based, including sample sizes, spatial and temporal scope of the measurements, sex, reproductive condition, and age classes or life stage are often not reported, providing few mechanisms for error checking and improvement. The outcome is that these species-level trait values become operationally static the moment they are published. A more effective approach would link standardized metadata about specimens, observation and measurement processes, and trait terms explicitly built to apply to individuals. Such an approach not only enhances discoverability and replicability of data but also facilitates research examining variation in traits across scales.

An improved system for communicating and storing traits reported at the individual-level is needed, where users can access open trait data and metadata and summaries of trait values can be dynamically generated. Building such a system need not start from scratch; we can learn from, and build upon, the infrastructure of open-access specimen databases and specimen data repositories such as (iDigBio: https://www.idigbio.org), VertNet (Constable et al., 2010; VertNet: https://vertnet.org), PaleobioDB (PBDB: https://paleobiodb.org), NOW (NOW database: https://nowdatabase.org/now/database), and Neotoma (Williams et al., 2018; NeotomaDB: https://neotomadb.org). These repositories have shown great success developing a community of data publishers and users built around adherence to community data standards that define key terms about collecting events, occurrences, taxonomies, and, if applicable, ways to define time. Researchers know what these key fields mean because they link to permanent definitions with examples [e.g., Darwin Core (dwc); Wieczorek et al., 2012] or are defined in the database schema. Standards are particularly essential for enabling research across disciplines, time periods, and spatial extents, providing a lingua franca that allows articulations across disciplines (LeFebvre et al., 2019). For example, standards and robust metadata fields are needed to aggregate data that spans time: zooarchaeological and paleontological specimens are collected at one date but lived at another and, thus, it is critical to properly report temporal context information.

Despite the enormous growth in specimen-level digital data, the biodiversity informatics community has paid much less attention to standardizing how traits measured from specimens are assembled and reported. This significant infrastructure gap has impeded broader integration and development of the extended specimen concept, where specimens sit at the center of a growing constellation of specimen-derived data (Lendemer et al., 2020). This gap is particularly important to close because trait data are already streaming into repositories, yet remain effectively undiscoverable and unusable (Troudet et al., 2018). Guralnick et al. (2016) showed a significant amount of trait data, including external measurements such as body length and reproductive state information, are often published along with specimen records. These data, however, remain hidden in notes or “associated data” fields, because existing standards, and the data publication systems constructed on those data standards, are not built for making all trait data types discoverable.

Even when data can be harvested and re-assembled from these “catch-all” fields, the challenge remains to harmonize and standardize trait information in a way that supports the broadest usability. In particular, trait definitions can be ambiguous due to differing homology definitions, uncertainty in specifics of the trait measurements (e.g., at which points on a bone are traits measured), uncertain measurement units, and/or lack of information or illustration of the trait, as well as updates in technology that change protocols for measuring traits (e.g., direct measurement on bone via calipers versus measurement from a photograph or 3D reconstruction). Even after proper standardization, studies investigating traits across time or taxa are still not comparable, because sub-disciplines have different practices about *what* to measure. Modern ecologists, zooarchaeologists, and paleontologists often do not use overlapping and comparable traits. For instance, modern mammalogists often take soft-tissue measurements, such as ear length and hindfoot length (e.g., Patton et al., 2000; Simmons and Voss, 1998; Voss et al., 2001), whereas zooarchaeologists and paleontologists take skeletal measurements in the absence of any soft tissue. Thus, both aligned trait and measurement definitions as well as analytical approaches for examining allometries are needed to help with linking and scaling across traits. Ontologies that leverage individual-level observations can help alleviate these issues (Walls et al., 2014). By creating ontology terms that are specific and nested, related terms can be mapped together, creating a unified terminology and supporting data integration. Furthermore, these ontological approaches can increase trait discoverability and can complement statistical approaches that quantify scaling relationships.

We have developed the Functional Trait Resource for Environmental Studies (FuTRES; few-tress) in response to the rapidly growing need for individual-level trait data. FuTRES has a back end (maintained by FuTRES) and a front end (interaction with users) for data ingestion and extraction. The back end is maintained by FuTRES and comprised of data validation, triplification, reasoning, and an API (application programming interface). The front end involves user input: interaction with (Data S1) template terms, trait terms to put into the ontology, and the input of data to GEOME (Deck et al., 2017; GEOME: https://geome-db.org) before being put into the FuTRES datastore. Our datastore is based on graph-like relationships among specimens, traits, and data, where new entities can be added without disrupting the model. It is built on new and existing trait ontologies and data integration workflow that aim to standardize and streamline trait data publication through our template preprocessing toolkits and thereby improve downstream use for paleontologists, zooarchaeologists, and neontologists (see the data life cycle: Michener and Jones, 2012; Griffin et al., 2017). FuTRES seeks to weave together efforts in trait and specimen data management to overcome the limitations of species-level trait data while building critical linkages to existing digital specimen records from which other specimen-related data can be found. These include linkages to existing repositories using occurrence identifiers and future linkages to MorphoSource (Boyer et al., 2016). We further expand the utility of FuTRES by also providing toolkits in beta release (see Supplemental Information) for data standardization and data cleaning (flagged data).

**Figure 1:**
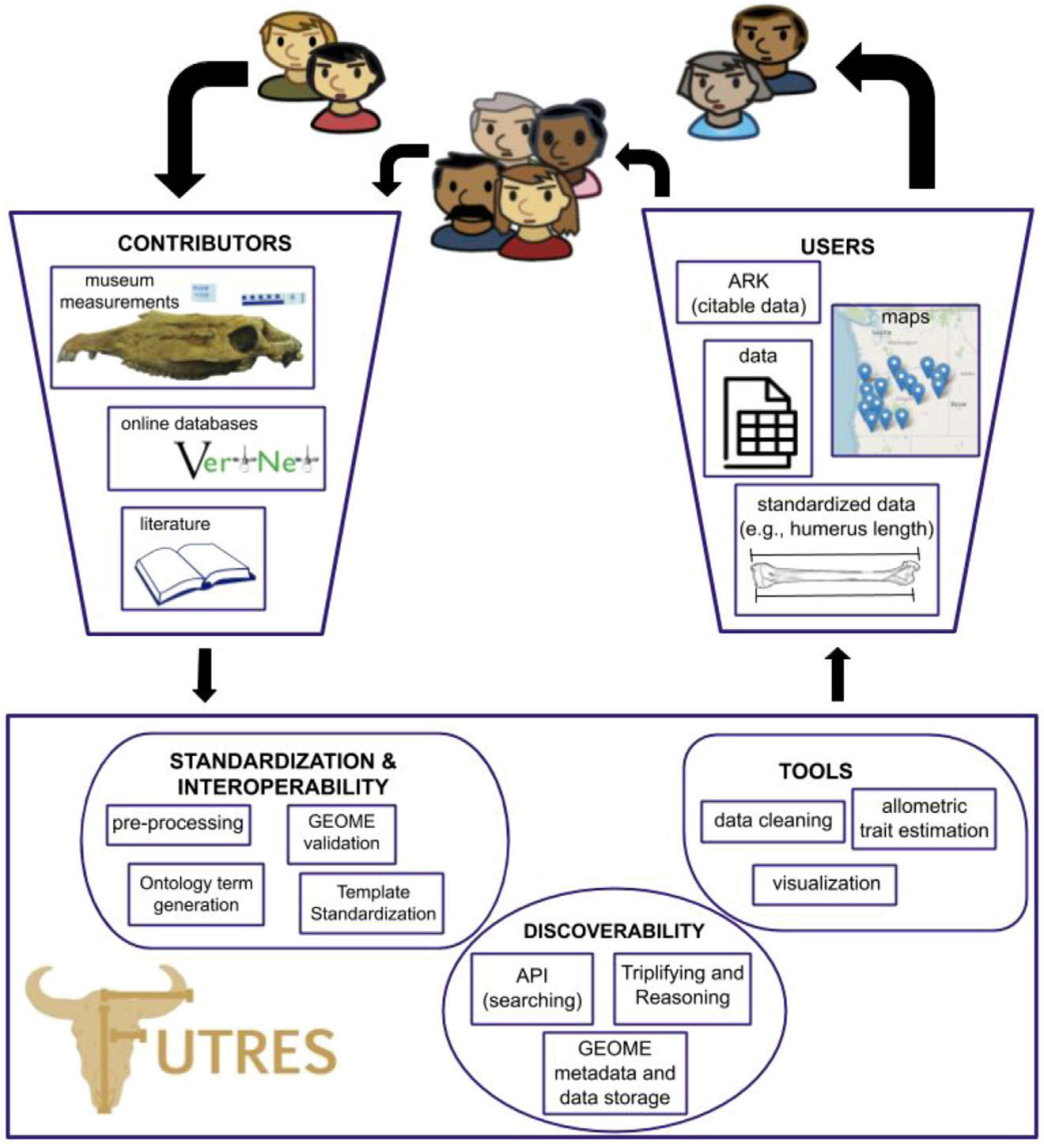
FuTRES data workflow The FuTRES community collects data from a variety of sources: the field, the literature, online databases, or from museum collections. The users input data formatted to a template accessed through GEOME, which accommodates paleo-, zooarchaeo-, and neontological metadata types. FuTRES works with the user to preprocess the data, but is also building tools, such as an RShinyApp (https://github.com/futres/RShinyFuTRES), that will allow submitters to prepare their own data for GEOME. The trait terms are defined and standardized; if a term does not exist, the user can create an issue to request a term through https://github.com/futres/fovt. The data are then validated and stored in GEOME. The FuTRES workflow then converts the data into RDF triples and reasons over the ontology and terms, resulting in standardized, discoverable data. The FuTRES team provides a cleaning routine for the data, filtering data, simple metrics about data, mapping and visualization of data, and ultimately the download of data. The user then can access and discover trait data at the specimen level.

FuTRES is currently focusing on mammalian trait data but will eventually support trait descriptions and measurements across the animal Tree of Life. The data contribution process for FuTRES is enabled via expansion of existing animal anatomy and trait ontologies, and it already provides access to millions of mammalian trait measurements via a data portal and API (Figure 1). We demonstrate how FuTRES facilitates access to specimen trait data and encourages community best practices for collecting and using these data. We showcase a user-requested, best practices-based data cleaning workflow for producing the best possible trait estimates, especially for the millions of neontological trait data measurement records that are already available but lack critical standardization for best use. We further provide two case studies to illustrate the benefit of using FuTRES to dynamically derive trait means and allometric equations for research relevant for modern as well as paleo- and zooarchaeological studies. The case studies showcase two common data uses: proper determination of distribution of body masses within a species, and predictions of body mass using skeletal material to predict potential body mass change over time.

## Results

### Developing FuTRES

FuTRES is a dynamic datastore connected to a community-available data ingest system, GEOME (Deck et al., 2017), which is an open-source toolkit that simplifies data import and validation for the community. FuTRES uses a specialized designed template in GEOME that defines required and optional fields for data uploads (https://github.com/futres/template). GEOME also provides means for providers to apply creative commons licensing, and embargoing data before release. The vast majority of records on FuTRES (>99.9%) are publicly available. A series of detailed help guides are available to support new providers getting started (https://github.com/futres/futres_website/blob/master/content/data_tutorial.md and https://github.com/futres/futres_website/blob/master/content/how_it_works.md).

FuTRES is a dynamic trait datastore, populated by pulling the most recent data loaded into GEOME and VertNet, annotating traits with updates from our FOVT (FuTRES Ontology of Vertebrate Traits; https://obofoundry.org/ontology/fovt.html) application ontology, so that each search retrieves the most up-to-date data available. In static datasets, data collection is paused at the time of publication; with FuTRES, an investigator can develop workflows such that each time analyses are run, the most up-to-date results are produced. Because the datastore is dynamic, we can better leverage the semantic web to link FuTRES trait data with other data sources, especially taxonomic resources to help update changing taxon concepts, but also environmental layers, gene sequences, and stable isotope records. This critical feature of FuTRES showcases how it can be part of the ecosystem of resources needed to implement the extended specimen concept (Lendemer et al., 2020).

The FuTRES datastore can be accessed via a simple web interface (FuTRES Datastore: https://futres-data-interface.netlify.app) or via an R package, rfutres (https://github.com/futres/rfutres). While current functionality of the R package is mostly focused on access to the datastore, it will also have functions for data cleaning, using the methods in this paper. Finally, in order to support those users who may want access to the whole of FuTRES, for larger analyses, we also provide a Zenodo archival snapshot that has a citable (https://doi.org/10.5281/zenodo.6569644; Gurlanick et al., 2022), and plan to produce those yearly for the community.

### Workflow and cleaning routine

We developed an extendable workflow based on a set of existing tools for taking unstandardized trait data reporting and converting them into formats that best enhance findability and accessibility of individual-level trait data. Using graph-like relationships allows for scalability because new data property terms and trait terms can be used without restructuring the workflow schema. For the first round of data ingest, we added 48 new ontology terms for the 12 traits (Table 1). These terms included anatomical terms, which will be a module in UBERON (Uberon Anatomy Ontology; obophenotype.github.io/uberon; Mungall et al., 2012; Haendel et al., 2014), as well as length terms, which currently are in the FOVT but will become available in OBA (Ontology of Biological Attributes; https://github.com/obophenotype/bio-attribute-ontology). FuTRES works with the community to develop new FOVT terms using a well-established mechanism for such requests (e.g., GitHub issues; https://github.com/futres/fovt/issues). With the workflow and ontology in place, seven datasets were standardized. Trait term requests can be made by creating an issue in the FOVT repository on GitHub. Standardized data are available through the FuTRES API and data portal (FuTRES Datastore: https://futres-data-interface.netlify.app).

**Table 1:** Summaries of traits ingested into FuTRES as of December 2020

Trait (IRI)	Synonyms	Records (non-modern)	Species
body mass (OBA:VT0001259)		196,098	2,357
body length with tail, *total length* (FOVT:0,000,001)	total length	525,733	3,755
ear length to notch (FOVT:0,000,005)	ear length external ear length	406,953	2,714
tail length (OBA:VT0002758)		473,211	2,854
pes length (OBA:1,000,048)	hindfoot length	469,877	2,789
forearm length (OBA:VT0010023)		19,346	614
astragalus lateral length (FOVT:0,000,013)	astragalus GLl talus lateral length	767 (722)	78
astragalus breadth (FOVT:0,000,021)	astragalus width talus breadth	733 (688)	76
calcaneus length (FOVT:0,000,022)	calcaneus greatest length calcaneus maximal length	308 (289)	48
calcaneus width (FOVT:0,001,079)		341 (311)	51
humerus length (OBA:VT0004350)		59 (45)	12
tooth row length (FOVT:0,000,030)		288	1
**Total**		**2,094,245 (336,746)**	**3,958**

Trait terms are the same as in the ontology (FOVT), with their IRI in parentheses. We also include counts for total number of records and for non-modern records. Synonyms for terms are either synonyms in the ontology or, in the case of the astragalus lateral length, the term we use in the paper to reflect terminology in von den Driesch (1976).

**Figure 2:**
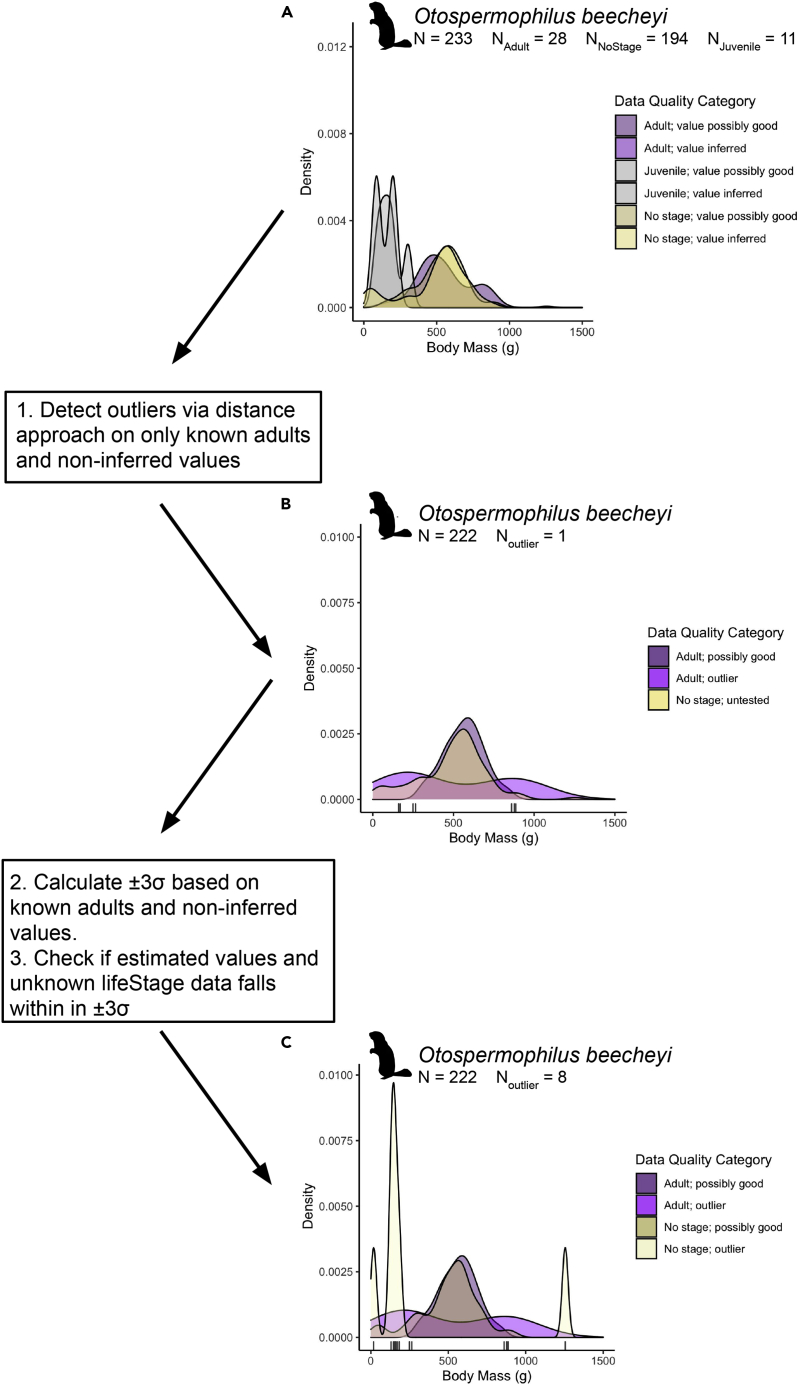
Data cleaning method with example (A–C). Here, we show *Otospermophilus beecheyi* as an example of the data cleaning process and success. Much data had unknown life stage (A), where purple colors denote known adults, yellow unknown life stage, and gray juveniles which we exclude from subsequent analyses. In this example, *Otospermophilus beecheyi* had 108 body mass records with no life stage reported. To remedy this, we created a distribution to test whether the unlabeled data were potentially adults. 1. Non-inferred, adult measurements were tested for outliers (results in B; gray bars below distributions are outliers). 2. From that set of data, we created +/−3σ upper and lower limits. 3. We tested the unlabeled, non-juvenile data against those limits (results in C; gray bars below distributions are outliers). Those within the limits we kept and labeled “possible adult; possibly good”, those outside of the limits were labeled “outliers” or “possible juvenile”.

We downloaded the ingested data from the FuTRES datastore, which has 3,958 species and 2,384,293 records. We then developed a cleaning routine to label outliers and potential juvenile records so that the data without known life stage are retained and enhanced (see example in Figure 2). We removed 56,993 records that were obvious outliers (2.5%). This point is highlighted in the example using *Otospermophilus beecheyi*, the California ground squirrel (Figure 2), and showcases how we were able to use data cleaning approaches to make previously unusable data usable by retaining records with unknown life stages [194 out of 233 records with unknown dwc:lifeStage; retained 222 records (28 known adults, 194 with unknown life stages but within adult body mass limits)]. The data cleaning toolkit checks whether values fall within the known adult distribution and flags the data as “possibly good, possible adult”, “outlier”, or “possible juvenile” in the “measurementStatus” column, letting the user decide whether they want to use it for downstream analyses. The data cleaning routine is rather liberal and biased toward keeping smaller trait measurements, and thus mean adult values may be slightly smaller than overall species body mass mean. Further cleaning, such as using known adult and juvenile body mass distributions, where warranted, may further help refine known body masses of both life stages, and we encourage community development of new efforts that can be implemented easily and linked to FuTRES. A key aspect of FuTRES is supporting and enhancing dissemination of these community-developed approaches, as well as helping to establish credit for such effort, such as publishing new protocols via protocols.io.

### Case studies

**Figure 3:**
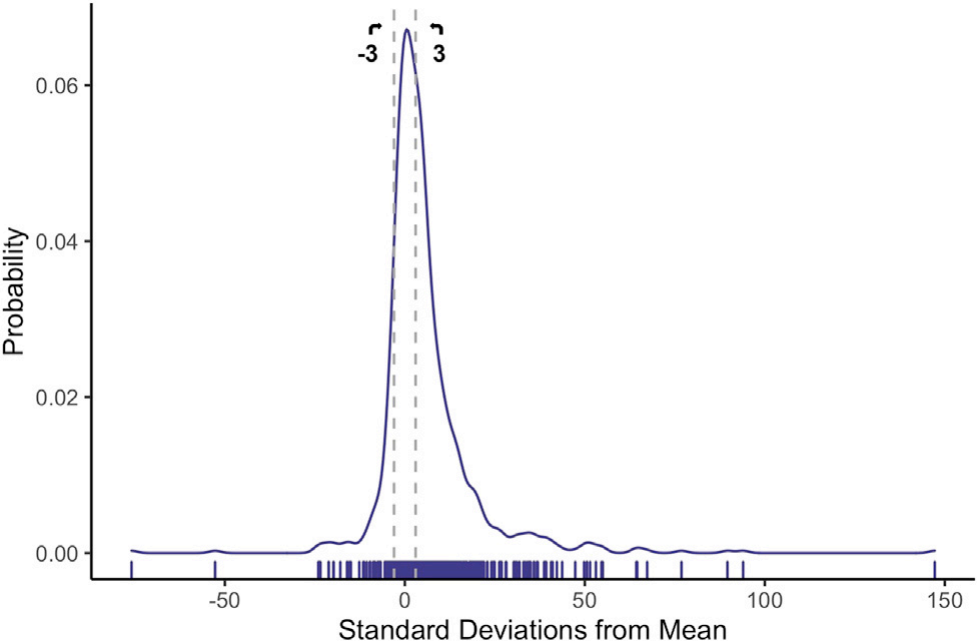
Differences between dynamic and static body mass estimates The distribution of the number of standard errors (*se*) of the PanTHERIA mean body masses (indicated by the vertical hash marks along the x axis) is from FuTRES average body mass. Dotted line (dark gray) indicates the +/−3 *se* from FuTRES average body mass.

In our evaluation of overall species means represented in the current FuTRES datastore, we find that reported species means from the literature (specifically PanTHERIA, Jones et al., 2009), while often not wildly far off, are also not generally in close agreement with species means in our dataset (Table 2; Figure 3). Only ∼32% of species mean body masses reported from PanTHERIA were within 3 standard errors (*se*) of mean values or within 95% and 5% quantiles of body mass by species in this study; ∼68% were not (Table 2; see also Table S1). Species means reported in PanTHERIA tended to be larger than the average body masses from our study (Table 2). We tested the relationships between sample size and the mean body mass difference to assure that sample size did not affect these results, where perhaps smaller sample sizes would result in a larger difference in body mass averages; however, we found no relationship (see also Table S2; Figure S1). Additionally, we tested for a relationship between body mass and difference in mean body mass, with the expectation that perhaps larger-bodied species with a wider body mass range would show a greater difference from mean body mass. We found a slight relationship, seemingly driven by an extreme case (see also Table S2; Figure S2), suggesting that sampling differences due to body size do not markedly affect this analysis.

**Table 2:** Comparison of mean species’ mass between PanTHERIA and this study

Group	N (%)	Within +/−3*se* (%)	Outside +/−3*se* (%)	>3*se* (%)	< -3*se* (%)
All	773 (100%)	244 (31.6%)	529 (68.4%)	422 (79.8%)	107 (20.2%)
<100g	559 (72.2%)	171 (30.6%)	388 (69.4%)	301 (77.6%)	83 (21.4%)
100–1000g	125 (16.2%)	44 (35.2%)	81 (64.8%)	64 (79.0%)	17 (21.0%)
1000–10,000g	46 (6.0%)	18 (39.1%)	28 (60.9%)	26 (92.9%)	2 (7.1%)
10,000–100,000g	31 (4.1%)	7 (22.6%)	24 (77.4%)	23 (95.8%)	1 (4.2%)

N percentages are out of total species. Percent of species within or outside of 3 standard errors (*se*) are compared to the sample size (N) for that group. More often than not, species means from PanTHERIA are outside +/−*3se* of the means calculated in this study. When they are outside of +/−3*se*, PanTHERIA tends to overestimate mean body size.

**Figure 4:**
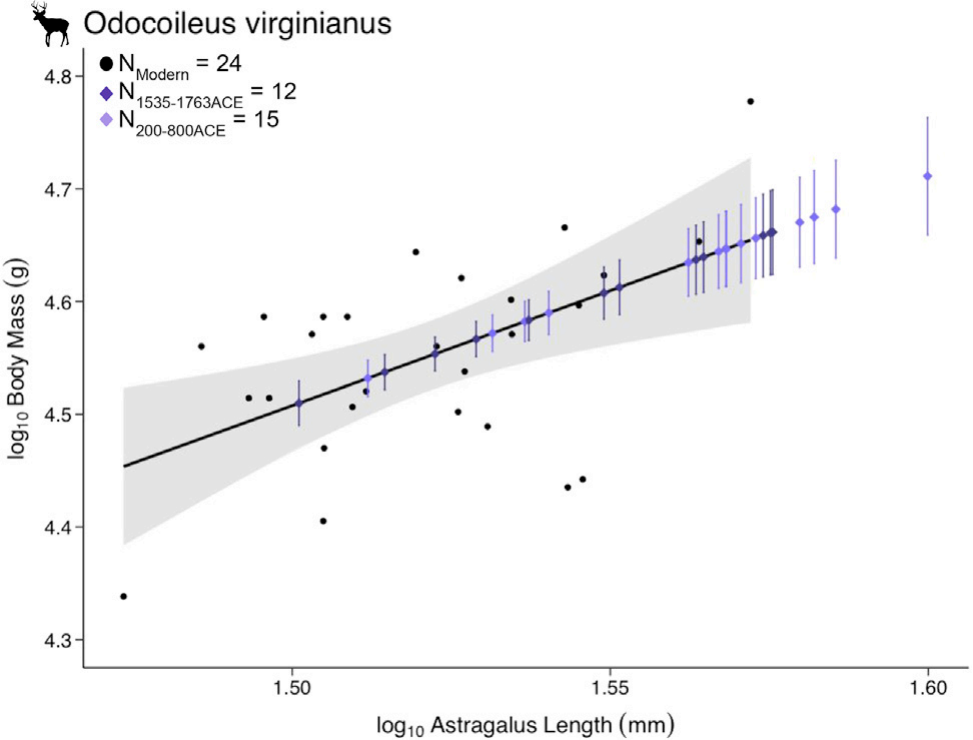
Body mass estimation for zooarchaeological deer astragali The relationship between modern deer astragali lateral length and body mass (black dots; black line) comes from data ingested to FuTRES from VertNet and K. Emery. Zooarchaeological data includes FuTRES data from K. Emery and additional data from Reitz et al. (2010). We predicted body mass (diamonds) from two sites (St. Catherines Island, 1565-1763 ACE in dark purple, and Fort Center, 200-800 ACE in light purple) and their associated +/− SE(vertical lines) from the relationship between modern deer astragali lateral length and body mass.

We predicted body mass for 27 specimens of *Odocoileus virginiaus* with astragalus length (Figure 4; see also Table S3). The greatest length of the lateral astragalus (GLl; von den Driesch, 1976; astragalus lateral length FOVT:00,000,013) measurements for the modern deer ranged from 29.75 to 37.33 mm (see also Table S4). Modern deer body mass ranged from 21.79 to 59.93 kg (see also Table S4). The allometric relationship is ${\log\;}_{10}\left(y\right)=2.04+1.45\cdot {\log\;}_{10}\left(x\right)$ with an *R*^*2*^ = 0.26 and p value = 0.004 (Table 3). The zooarchaeological astragalus measurements fell within the range of the modern deer (31.5–39.8 mm). Likewise, the resulting body mass estimates fell within the range of modern deer (32.3–51.4 kg; see also Table S3). We also estimated body mass using the constants of slope and intercept from the original lab calculations curated in the FM-EAP (see also Table S3). We tested whether the single-value estimated body mass fell within the range of newly calculated body mass within 2 *se* (95% confidence interval) calculated in this study (see also Table S3). We found that the original body mass estimates did not fall within the range of predicted body mass values from this study, often being underestimates of body mass.

**Table 3:** Constants for allometric equations for estimating the body mass of *Odocoileus virginianus* from astragalus lateral length measurements in FuTRES

	${\log\;}_{10}\left(a\right)$ (*se*)	b(*se*)	df	*Sample* *Size*	*R*^*2*^	p value
This study	1.45 (0.64)	2.04 (0.97)	25	27	0.29	0.004
Reitz (2008)	−6.79	5.29		10	0.87	

Constants and needed information, such as SE(*se*) of the slope (*b*), intercept (log_10_(*a*)) and sample size, are needed to estimate (log_10_(*y*)), which in this case is body mass. We show our revised intercept, slope, r-squared value (*R*^*2*^), and p value with degrees of freedom (df) for estimating body mass compared to those derived in the 1990s in the FM-EAP with a smaller sample size (unpublished data) and used in Reitz (2008).

## Discussion

Trait data resources have flourished in the past decade [Atlantic Mammal Traits (Gonçalves et al., 2018); BIEN (Enquist et al., 2016), TRY (Kattge et al., 2011), DISPERSE (Sarremejane et al., 2020); Maasri, 2019; Coral Trait Database (Madin et al., 2016); (fungaltraits: https://github.com/traitecoevo/fungaltraits); Meiri, 2018; (ButterflyNet Trait Database: https://butterflytraits.org); EltonTraits (Wilman et al., 2014); sFDVent (Chapman et al., 2019); FishTraits Database (Froese and Pauly, 2019); The Global Ants Database (Parr et al., 2017; Antworld: http://antworld.org); Sharkipedia (Kindsvater et al., 2020); COMBINE (Soria et al., 2021); AnimalTraits (Herberstein et al., 2022)], revolutionizing our ability to link evolutionary processes to how ecosystems function. However, many of these resources focus on species’ trait values, such as mean body mass or number of offspring. This focus limits our ability to link trait values to the specific environmental and ecological contexts in which they are observed. While some data are better than no data, there are key questions about quality, coarseness, and long-term usability of such compendiums. Even in cases where research communities have started capturing individual-level trait data, these resources often lack community collaboration and are not built on a strong foundation of trait semantics and open data principles. Conversely, FuTRES is a community-developed and ontologically robust trait datastore with an initial focus on mammals that is extensible broadly to the Tree of Life.

FuTRES relies on widely used trait ontologies and is synchronized with the existing, well-developed data ingestion pipeline (Stucky et al., 2018). The power of ontology and this workflow is 2-fold. First, as more trait terms are added, the ontology will become more flexible both in trait specificity and generality, enabling trait discovery. Second, our workflow (Walls et al., 2014; Stucky et al., 2018) connects instances of a specimen occurrence to instances of a specimen measurement. The metadata and ontological terms easily connect with other data repositories that use specimen occurrences, such as the Global Biodiversity Information Facility (GBIF; https://www.gbif.org). This facilitates encoding data values (measurementType hasValue isNumeric) and units (measurementValue hasUnits isString) into the ontology, increasing interoperability. These standardization tools also reduce the need to wrangle data [an estimated 80% of data handling time (Furche et al., 2016)], facilitating research by centralizing standardization of datasets that would otherwise be cumbersome or impossible to accomplish by individual actors.

To further increase data usability, best practices for error checking and data cleaning are incorporated into the FuTRES cleaning routine (Figure 2; see STAR Methods). We emphasize keeping verbatim fields and flagging data so that no information is lost or modified in existing data columns. Our data cleaning routine did reasonably well at removing putative outliers and providing a way to filter for adult body mass records. The cleaning routine we present here is conservative, often retaining the lower (smaller) end of measurement, which may represent erroneous data or unlabeled juveniles. Still, our dynamically derived mean trait values are, generally, close to means reported in the literature. Sometimes, the conservative data cleaning resulted in mean body masses lower than PanTHERIA and the Animal Diversity Web (ADW; Myers et al., 2020; https://animaldiversity.org). In the case of *Microtus californicus* (n = 3,004), the California vole, our average body mass (38.0 g) was lower than PanTHERIA (57.4 g), yet still within the range provided by ADW (38–108 g). By contrast, for *Myodes rutilus* (n = 15,334), the northern red-backed vole, we retained lower body mass estimates, and still the mean body mass (22.2 g) was greater than PanTHERIA (19.9 g) but well within the range of reported body mass from ADW (20–40 g). In both cases above, the sample sizes in FuTRES are thousands of individuals—even with potential juvenile bias—and so the mean body mass likely reflects the actual species’ mean body mass better than the estimates in PanTHERIA and ADW. The benefit of the FuTRES data is that sample size is known, and each report is tied to specimen records and specimens, providing a researcher with the most information possible to make judgments about usability of the data.

These cases of conflict with other trait resources highlight one of the important benefits of FuTRES: factors that influence mean trait values, such as sample size, geographic range, age, and sex, are *known* and can be explicitly accounted for in downstream analyses. Access to all of the underlying specimen-level data allows researchers to make informed decisions about the quality of summary statistics. For instance, a significant difference in average body masses between PanTHERIA and FuTRES may be seemingly unimportant if the difference is small: for *Pteronotus davyi*, a small bat, the average body mass differs by 25 *se*, equating to 2 g. This amount may seem trivial, but it represents ∼27% of the species' total body mass. These small differences are therefore non-trivial, with impact for inferences about species life history that may vary across space and time.

FuTRES focuses on multiple traits, often collected from the same organism, providing another significant advantage compared to many trait compilations. Reconstructing body mass is a common step in paleo- and zooarchaeological research because so many other life history traits are known to depend on body mass (Hopkins, 2018; Damuth and MacFadden, 1990; Schmidt-Nielsen, 1975). Having a large sample size of modern skeletal and body mass measurements improves reconstructing body mass of paleo- and zooarchaeological specimens. Access to datasets where skeletal measurements and body mass have both been reported allowed us to show the power of a dynamically derived allometric equation for reconstructing body mass in white-tailed deer (*Odocoileus virginianus*) from archaeological specimens. Our case study showcases how using multiple data resources from both neontological and zooarchaeological collections can refine body mass estimates and link across temporal scales. However, it is worth noting that FuTRES — and other datastores — often lacks body mass measurements for large-bodied animals, and when these are present it is not always clear what state the animal was in (e.g., skinned, gutted, and preserved) when measured. Body mass measured prior to, and after, viscera are removed is dramatically different, and reconciling body mass data when reporting about preparation methods can be sparse remains challenging.

While efforts to continue collecting and reporting large mammal body mass data and metadata are needed, FuTRES provides a useful means to assess data gaps and prioritize needs based on community input. Furthermore, we note the value of directed work with citizen scientists and land managers, who could help alleviate gaps in assembling body mass data for animals taken by legal hunting or culled by government land management programs. In addition to citizen science work and land manager contributions, a best practice for all field biology is to have a procedure to take body masses of animals that are sampled (live or dead) so we can begin building more extensive large mammal datasets. In the interim, Saarinen et al. (2021) suggest an approach to choosing the optimal body mass estimation regression from legacy regressions that are currently available in the literature. The authors compared the percent error of the body mass estimate for each skeletal or dental element of wild *Equus* to determine the best predictors for body mass. Because of the dearth of body mass data on extant large mammals, these methods are important not only for paleo- and zooarchaeological body mass reconstructions but also for estimating the body masses and body condition indices of modern large mammals, such as zebras, collected over the last two centuries.

FuTRES exists to streamline and automate the process of assembling and integrating biological trait data measured from individuals, facilitating the use of trait data in a similar way to that pioneered for genetic data by GenBank (Benson et al., 2012). FuTRES supports data producers in sharing their data and connecting to data users, with a focus on community development and best practices. The coauthors of this paper, who were active participants in workshops (https://futres.org/workshop) and post-workshop activities, are just the first step of a growing research community with strong interest in understanding the basis of phenotypes and phenotypic variation. As FuTRES matures as a data resource, researchers will be able to collect and share data more easily, and in ways that instill best practices both in data collection and reporting, while also providing a crucial means for data discovery and use that can facilitate testing new hypotheses about trait evolution in novel and unexpected ways. We call out the particular importance of linking from intra- to interspecific trait variation at the broadest scales (Read et al., 2018). Finally, tools in FuTRES allow easy tracking of the use and re-use of trait data, so field researchers can clearly document the impact of their collecting efforts, justifying funding and institutional support for new fieldwork and maintaining collections.

While the initial focus for FuTRES has been on linear measurements of mammals, the ontology structure of the data resource allows it to be expanded to handle any kind of trait for any kind of organism. There are efforts underway, for example, to add non-scalar traits to FuTRES, some of which represent ecological interactions, like shark bites or parasite load. In particular, we are exploring the expansion of measurement data from the current focus on legacy linear morphometrics to the growth area of 3D geometric morphometrics (Hernández et al., 2017) and describing landmark locations using trait semantic approaches. With the use of the FOVT application ontology, the path is already laid to begin adding trait data for other vertebrates beyond mammals, and we hope that larger communities working across the Tree of Life coalesce around individual-based trait repositories. We close by noting that FuTRES is not simply meant to be an archive of trait data, but rather a growing repository where new tools, such as the R package, rfutres, and knowledge can grow. As a final example, FuTRES is actively exploring assembling real-time allometry equations that change as new data are assimilated into the datastore and cleaned for use and providing these outputs such that the links to the data used are persistent. This approach reflects a vision of a knowledge resource that is focused around community-established best practices.

### Limitations of the study

FuTRES is still in development, and so does not yet accept all types of trait data. We have concentrated on 2D linear measurements of mostly mammal appendicular elements, as reflected in our case studies. We encourage readers and future data users and contributors who wish to suggest linear measurements to submit a term request via a new issue at https://github.com/futres/fovt.

Our case studies showcase both the power and some potential limitations of individual measurements from specimens. For example, lack of reporting of life stage, which is surprisingly common in published specimen records, can make assessment of adults versus juveniles difficult and subjective, limiting use. In general, improved reporting of specimen-level metadata will increase usability downstream for research. To overcome this challenge, we built reusable cleaning routines that will be made available in the next version of the R package, rfutres, (https://github.com/futres/rfutres). These can be refined further, as they likely retain some reporting of juvenile trait values. We encourage community development of enhanced methods by submitting issues on GitHub. Still, the routine provides a set of best practice approaches for cleaning datasets, including flagging data so that users can make informed decisions about data quality.

Finally, we note that our body mass comparison case study focused on a single, highly curated source (Jones et al., 2009). We are aware that there are other compilations, potentially of high quality, such as the Animal Diversity Web, that differ from both this study and Jones et al. (2009) mean estimates of body mass. Our goal is not to do a comprehensive comparison of estimates across resources but to show the power of being able to easily assemble body mass distributions built from individual-level reporting, which underlies creating any mean body mass estimates.

## STAR★Methods

### Key resources table

**Table:** 

REAGENT or RESOURCE	SOURCE	IDENTIFIER
**Deposited data**		

FuTRES Data	Guralnick et al., 2022	Zenodo Data: https://doi.org/10.5281/zenodo.6569644
Discovery Environment	CyVerse	Raw data files: https://user.cyverse.org

**Software and algorithms**		

Code used in paper	This paper	https://github.com/futres/Best-Practices/releases/tag/v2.1
fovt-data-pipeline	This paper	https://github.com/futres/fovt-data-pipeline
Rfutres	This paper	https://github.com/futres/rfutres/releases/tag/v1.0.0
Traiter		https://github.com/rafelafrance/traiter
OutlierDetection		https://CRAN.R-project.org/package=OutlierDetection

**Other**		

FOVT (FuTRES Ontology of Vertebrate Traits)	This paper	https://github.com/futres/fovt

### Resource availability

#### Lead contact

Requests for information or resources should be directed to the Lead Contact, Meghan A. Balk (meghan.balk@gmail.com).

#### Materials availability

There were no specialized materials used for this study.

### Method details

The following are steps towards creating a cohesive, sustainable, individual-based trait datastore that supports a community of users who can publish and access content (see our tutorial: https://futres.org/data_tutorial). First, we developed a backend, FuTRES-maintained workflow to standardize metadata and trait terms and support trait data publication. We then populated key legacy datasets that span from smaller, single-study datasets to millions of extracted traits from aggregators such as VertNet (Constable et al., 2010; https://vertnet.org), representing data from both modern and deeper time contexts. The initial datasets were ingested by the FuTRES team, with the goal of future datasets being uploaded by the community of researchers generating these data. We also created an outline for best practices in data cleaning, in an effort to preserve data that may otherwise be removed during later data filtering steps. From this cleaned set of data, we compare derived mean trait values to those in species-level databases that have been assembled based on literature. This comparison demonstrates the value of specimen-level data storage and integration efforts.

#### Data collection

An impetus for FuTRES was to make accessible trait data that arealready available but lack standardization or are effectively hidden in current published datasets. Through the FuTRES team and our initial FuTRES workshop in summer 2019 (https://futres.org/workshop2019), we amassed and integrated into the FuTRES datastore seven mammalian species metric datasets (Table 1). Besides new VertNet data, the modern data also include smaller datasets of *Puma concolor* (cougar) weight (intact, skinned, and gutted) and total length from Oregon Department of Fish and Wildlife (2020), *Odocoileus virginianus* (white-tailed deer; K. Emery) from Georgia and Florida with intact body mass and various post-crania skeletal measurements from von den Driesch (1976), *Otospermophilus beecheyi* (California ground squirrel; Blois et al., 2008) from California with soft tissue measurements, body mass and toothrow length, and *Aepyceros* (impala; A. Villaseñor) from east Africa with various cranio-dental and post-cranial measurements following von den Driesch (1976). White-tailed deer and California ground squirrel datasets contain a mix of whole carcass measurements and skeletal measurements, allowing for linkages between traits. The zooarchaeological datasets include two archaeological datasets on *Odocoileus virginianus* (white-tailed deer): one from the Florida Museum Environmental Archaeology Program (FM-EAP) collections, which was ingested into FuTRES, and one from Reitz et al. (2010) (Table S3). A key paleontological resource is a database of over 20,000 records of fossil Equid specimen-based cranio-dental and post-cranial measurements following Eisenmann (1988; Bernor et al.,. 1997) from R.L. Bernor with a global distribution spanning 16 mya to recent. The paleo- and zooarchaeological datasets are heavily curated with large numbers of skeletal trait metrics. Together, these datasets encompass 3,958 species, over two million measurement records, and 12 traits (discussed below; Table 1), with more traits to be added. The original data is stored in the CyVerse Discovery Environment (https://de.cyverse.org). Below we describe how these datasets were ingested into FuTRES and show their value and utility for enabling new research.

#### Back end

##### Workflow

The FuTRES data processing workflow improves interoperability of datasets by standardizing metadata and trait names to ontologies and data standards (Figure 1). We built upon an existing ingest pipeline (Stucky et al., 2018) by modifying it for vertebrates and for three intersecting disciplines (paleo-, zooarchaeo-, and neontology). The workflow includes four steps: preprocessing, converting the data to RDF-OWL triples, reasoning (inferring additional facts based on the ontology), and exporting to a semantic toolkit, GEOME (Genomic Observations MetaDatabase; Deck et al., 2017; https://geome-db.org), which tracks metadata and validates datasets. Here we focus on preprocessing, because the other steps remain largely unchanged from Stucky et al. (2018). Preprocessing includes identification of the minimum set of metadata terms required for paleo-, zooarchaeo-, and neontology, standardization of column headers, and standardization of trait terms. The pre-processing steps below cover existing datasets requiring conversion and transformation before proceeding to the additional processing steps. Data sets can also be submitted directly to GEOME using the FuTRES Sample Project Template Generator, which automatically creates a datasheet with the required fields and their definitions, therefore lessening the need for pre-processing. We have a tutorial for data uploading available online (https://futres.org/data_tutorial). We additionally made a web application (in beta; https://github.com/futres/RShinyFuTRES) to re-format legacy datasets so that they are able to be uploaded into GEOME.

##### Template

All datasets require a minimum amount of metadata (e.g. a title, description, ownership). After capturing these dataset level metadata, data were mapped to a template that standardized column headings and data types to ensure reproducibility and facilitate creation of RDF triples (Figure 1). We decided which columns (i.e., metadata) to include through consultation with a group of disciplinary experts during the summer 2019 workshop as well as with specific data providers (Data S1). We encourage the use of uniform resource identifiers (URIs) linking to associated data whenever possible. The template requires the minimum information needed for trait data to be usable by researchers across disciplines and has the option for discipline-specific fields, such as the newly developed Darwin Core chronometric extension for paleontological and zooarchaeological temporal data (see https://github.com/tdwg/chrono), as well as FuTRES-specific cultural context metadata terms useful for defining zooarchaeological data. We created terms for unique identifiers that track the measurement event (diagnosticID) to the specimen (materialSampleID) to the individual (individualID) so that multiple measurements on the same specimen or individual can be associated. Required values are shown in Data S1. All field names follow the structure of (camelCase; definitions) and use terms from Darwin Core (Wieczorek et al. 2012), when available.

##### Ontology

To standardize trait terms, we used UBERON (Uber-anatomy ontology; Mungall et al., 2012), the species-neutral ontologies for animal anatomy, and OBA (Ontology of Biological Attributes; Dönitz and Wingender, 2012) for traits. Because the timing of their release schedules would delay our addition of new terms to these ontologies, we have created trait terms we need in an application ontology, the FuTRES Ontology for Vertebrate Traits (FOVT; https://obofoundry.org/ontology/fovt.html). The FOVT trait classes will be replaced by OBA terms as soon as they are released. The hierarchical arrangement of trait ontology terms allows for flexibility and integration across taxa and disciplines that measure traits differently. For example, if “humerus length” is the measurement of interest, the ontology allows for differing degrees of specificity. One could select known specific endpoints for humerus length (trochlea to caput; trochlea to ventral tubercle, etc.). If a data curator does not know which specific term to use, or if the researcher extracting the information is only curious about general measures of “humerus length” across taxa, then the general term “humerus length” can still be used. Because of the nested hierarchy, a search on the general term will return humerus lengths for all the ways it is measured. This allows the data captured to be both precise and flexible for the user and contributor.

##### Data validation

Once the data are processed and standardized, they are uploaded and validated in GEOME. In GEOME, researchers can access the template (described above) and/or uploaded data. Data validation in GEOME reports validation errors to data submitters and helps users fix their data. GEOME and VertNet data are then aggregated and processed using a data processing workflow (https://github.com/futres/fovt-data-pipeline) which performs final validation steps, triplifies, reasons, and then loads reasoned data into a document store (ElasticSearch). Data reasoning is computed using the ontology-data-pipeline codebase (https://github.com/biocodellc/ontology-data-pipeline), which is run as an available Docker container and draws on FOVT. After data are validated, integrated, and reasoned, the pre-reasoned data are loaded into an ElasticSearch database where data are made available to researchers through the FuTRES website and an API (application programming interface), where researchers can visualize taxonomic and trait coverage. FuTRES data resources are also available via a prototype web portal that provides a simple faceted search approach for filtering by species, datasets and traits of interest.

##### Data cleaning

We developed a prototype data cleaning toolkit, first applied to body mass, body length, and tail length, but usable for all measurement traits. This cleaning toolkit is especially valuable for cases of automated trait extractions from heterogeneous reporting such as in the VertNet dataset, where trait values may either be misreported in the original record or assembled improperly during automated extraction from *Traiter* (https://github.com/rafelafrance/traiter). A key goal of the data cleaning effort was to provide a means to help users find and filter the most credible reports of adult trait values. This required both flagging improbable values and determining whether records without life stage reporting could be inferred as adults (see example in Figure 2, panel A). We developed an R-based (R Core Team, 2018) workflow to check for outliers on the full dataset. To accomplish this, we create a column, "measurementStatus" to report if the datum is an "outlier", if there are "too few records" to check, or if it is a "possible juvenile". First, we check whether a species has at least 10 records (otherwise labeled “too few records” in measurementStatus). The workflow starts with a Mahalanobis Distance outlier test using the package OutlierDetection (https://CRAN.R-project.org/package=OutlierDetection) in R for known adults where body mass units were recorded (i.e., non-inferred values), which is used in the case studies (below; Figure 2, panel B). From the new distribution, which includes only adults and excludes extreme outliers likely to be mistaken trait values from automated assembly and inferred values, we test if the distributions of trait values are normal, log-normal, or not (in column "normality"). For those that are normally or log-normally distributed, we calculated an upper and lower limit (columns "upperLimit" and "lowerLimit") range based on 3 standard deviations (σ) from the mean (±3σ) and record the method as "sd" or "log sd" for standard deviation method in "upperLimitMethod" and "lowerLimitMethod". For those that were non-normally distributed, we calculated upper 95% and lower 5% quantiles (with method defined as "quantile"). We then reassessed values without labeled life stage against our empirically determined upper and lower limits (Figure 2, panel C). We labeled those records with trait values outside the upper as "outlier" and lower as "possible juvenile" in "measurementStatus". Those values within the upper and lower limits were labeled "possible adult; possibly good". As discussed below, this is a conservative method for flagging records, allowing data users to further develop their own customized cleaning approach. This cleaning routine will be made available as part of the next release of the FuTRES R package, rfutres (https://github.com/futres/rfutres).

We uploaded the raw, unstandardized data as a means to keep a “before processing” archival version in the CyVerse Discovery Environment (https://de.cyverse.org) in the Data Commons, with a permanent link to the data. The template and standardized pre-processed datasets are available on GEOME under the "FuTRES" project. FuTRES' intention is for data replication to be possible post-extraction from the FuTRES API (https://futres-data-interface.netlify.app). The data provider can download a template from GEOME and standardize and validate their data through GEOME. Additionally, data providers are encouraged to add metadata under their project and expeditions (i.e., datasets) in GEOME. Data extracted from GEOME will link back to the project under GEOME (see columns expeditionCode and projectId). The datastore download and VertNet version ingested into the datastore are available on the CyVerse Discovery Environment (DOI pending).

#### Front end

##### User input

To relieve bottlenecks in the workflow, we are creating an R Shiny App to help users manipulate and transform their data into a format compatible with the FuTRES template. The R Shiny App has the following functions: rename columns, check that all required columns are there, transform data from short-form to long-form, remove any "measurementValues" that are "NA", standardize various columns such as dwc:locality, dwc:yearCollected, and dwc:materialSampleType. The functions in the R Shiny App are the most common transformations done on the data ingest for this study.

Once the user has data that is formatted, they can upload it to GEOME (following the data tutorial on our webpage: https://futres.org/data_tutorial). First, they create a project under the FuTRES Team. We encourage users to write an abbreviated abstract about the data being uploaded. We use the naming convention "FuTRES_taxon_contributor_locality_time.period_version or date" for each dataset (called an expedition in GEOME). The user can then upload and validate their data (discussed above). This will then be pulled into the workflow (discussed above).

##### API and R package

FuTRES datastore has an API (https://futres-data-interface.netlify.app) that allows users to search and download the data available. We have also created an R package called rfutres (https://github.com/futres/rfutres). The package has functions for downloading the entire datastore (example in the readme file) and filtering for a refined set of data.

### Quantification and statistical analysis

#### Case studies

Two case studies were conducted to exemplify the process by which large datasets of individual level trait data can be analyzed to quantify differences relative to traditional, species-level trait dataset reporting. The first case study compares body mass summaries from the FuTRES datastore to those from published literature based on species-average body masses. The second case study focuses on the temporal dimension of trait data by providing white-tailed deer body mass estimates derived from pre-Hispanic, Colonial, and modern skeletal elements.

(1) *Comparison of mean body mass values*

We compared the means of previously published species-level body masses from PanTHERIA (from published literature; Jones et al., 2009) to the means of the individual-level body mass data from the cleaned, full dataset of body mass estimates in FuTRES. We matched the datasets by species, resulting in 108 shared species. We calculated a one-sample Student’s *t*-value for each difference between these means as:

(Equation 1) $$t_{mass}=|\left(body\;mass_{PanTHERIA}-body\;mass_{FuTRES}\right)÷\left(se_{FuTRES}\right)|$$

where *se* is the standard error. Any difference that was greater than the critical *t*-value given the degrees of freedom for a species sample was considered significantly different. We further performed a Benjamini and Hochberg (1995) correction because p-values were calculated repeatedly. We tallied the number of species where the PanTHERIA body mass was over ±3 *se* from the average FuTRES datastore mean body mass.

(2) *Comparison of body mass estimates*

The second case study showcases how FuTRES can be used to generate allometric equations that allow for prediction of uncertainty in the reconstruction of inferred values, such as body mass, for fossil or zooarchaeological specimens. In the past, these inferred values have often been calculated from equations from key works such as in the chapters in Damuth and McFadden (1990), but the equations published in these legacy studies do not provide key information about error estimation. Consequently, it has become too common for researchers to present inferred values with no uncertainty (Hopkins, 2018). Here we selected *Odocoileus virginianus*, white-tailed deer, which have both whole-body live body mass and astragalus lateral length measurements (Table S4). This dataset yielded measurement of live body mass and astragalus lateral length [astraglaus GLl (von den Driesch 1976)] of 30 modern *O. virginianus* specimens from the Florida Museum Environmental Archaeology Program (FM-EAP) collections. Astragali were selected for body mass estimates because they are weight bearing elements shown to correlate well to body mass and are well preserved in archaeological assemblages (Wolverton et al. 2007). We also selected astragalus lateral length measures of 27 zooarchaeological *O. virginianus* without body mass measurements. All zooarchaeological specimens are from Florida and Georgia, including specimens from Fort Center (8GL13, occupied 200-800 ACE; date from Sears 1982) and from the Mission and Pueblo of Santa Catalina de Guale, St. Catherines Island (9Li13 and 9Li8, occupied 1565-1763 ACE, date from Reitz et al., 2008), with metrics from Reitz et al. (2010) are curated at the FM-EAP.

We calculated an allometric equation relating astragalus lateral length (GLl, von den Driesch 1976; talus lateral length FOVT:00000013) to body mass with standard errors representing a known range of variation in allometric correlation:

(Equation 2) $${\log\;}_{10}\left(y\right)={\log\;}_{10}\left(a\right)+b\cdot {\log\;}_{10}\left(x\right)$$

where a is the y-intercept, b is the slope (Huxley and Teisser 1936).

The equation was applied to zooarchaeological specimens with known astragalus lateral length (GLl, von den Driesch, 1976; talus lateral length FOVT:00000013) to reconstruct body mass. Specifically, by knowing the sample size (*n*), mean ($\bar{x}$), standard error of the slope ($se_{b}$), and standard error of the residuals of the line ($se_{y\cdot x}$), we can produce a mean and standard error of body mass estimates for each specimen with known astragalus lateral length and unknown body mass. We used the following equation (Zar, 1999) to calculate the standard error for reconstructed body mass:

(Equation 3) $$se_{\hat{y}i}=\sqrt{{\left(se_{y\cdot x}/\sqrt{n}\right)}^{2}+{\left(se_{b}\cdot \left(x_{i}-\bar{x}\right)\right)}^{2}}$$

We compared the range of body mass estimates to the body mass estimate derived by the FM-EAP lab in the early 1990s based on 10 FM-EAP modern deer. The constants of intercept $\left({\log\;}_{10}(a\right)$ = -6.71) and slope (b = 5.29) for body mass, with an *R*^*2*^ value of 0.87. We evaluate body mass differences between this study and the established formula using the Zar (1999) based method.

## Data Availability

• Raw original data are available through the CyVerse Discovery Environment, with permanent, publicly-accessible links in the scripts, with only a free account required (https://user.cyverse.org). All standardized datasets are available at GEOME under the FuTRES project. The version of VertNet and the download from the FuTRES datastore are available in CyVerse. Finally, an archive of all current FuTRES data that are publicly available can be found at Zenodo (Zenodo Data: https://doi.org/10.5281/zenodo.6569644; Guralnick et al., 2022). • Scripts for data cleaning and analyses are available at https://github.com/futres/Best-Practices (v2.1; https://github.com/futres/Best-Practices/releases/tag/v2.1). Code for the rfutres package is available at https://github.com/futres/rfutres/releases/tag/v1.0.0. • Any additional information required to reanalyze the data reported in this work paper is available from the lead contact upon request.

## Supplemental information

Document S1. Figures S1 and S2 and Tables S2 and S4, and Data S1

Table S1. Comparison between our mean mass and PanTHERIA mean mass, related to Table 2 and Results, Case studies From the FuTRES dataset of species body mass that overlapped with PanTHERIA, we calculated sample size (N), minimum mass, maximum mass, and average mass. The "% Different" is the difference between the two average masses, divided by the FuTRES average. The critical *t* value is the minimum number of standard errors (*se*) that the species’ average mass from PanTHERIA must be from the species’ average mass values from the FuTRES datastore for a significant difference (p-value less than 0.05; in bold). We applied a Benjamini and Hochberg (1995) correction to the p-values. Bolded corrected p-values are for PanTHERIA masses that are significantly different from FuTRES average mass. See Table 2 and Results, *Case studies*.

Table S3. Predicted body mass values (g) from zooarchaeological astragalus lateral lengths (mm), related to Figure 4, Results, Case studies, and STAR Methods We compare our allometric equation with standard errors for mass estimates to estimated mass derived by the FM-EAP lab in the early 1990s based on 10 FM-EAP modern deer and used in Reitz (2008). Those calculations included constants of intercept (-6.71) and slope (5.29) for body mass, with an R-squared value of 0.87 (FM-EAP unpublished lab records). Those mass estimates which lie within ±2 standard errors [se; upper (+2*se*) and lower bound (-2*se*)] of predicted body mass from our equation are denoted by TRUE. The mass regression was calculated in log-log space, so the SE is in log_10_(g). See Figure 4, Results, *Case studies*, and STAR Methods.

## Footnotes

Supplemental information can be found online at https://doi.org/10.1016/j.isci.2022.105101.
