# Supplementary material for: A solution to the challenges of interdisciplinary aggregation and use of specimen-level trait data
Source: iScience. 2022 Sep 13;25(10):105101. doi: 10.1016/j.isci.2022.105101 (PMC9535407; doi:10.1016/j.isci.2022.105101)
Supplement: Document S1. Figures S1 and S2 and Tables S2 and S4, and Data S1 [file mmc1.pdf]

iScience, Volume *25*

Supplemental information

A solution to the challenges of interdisciplinary aggregation and use of specimen-level trait data

Meghan A. Balk, John Deck, Kitty F. Emery, Ramona L. Walls, Dana Reuter, Raphael LaFrance, Joaquín Arroyo-Cabrales, Paul Barrett, Jessica Blois, Arianne Boileau, Laura Brenskelle, Nicole R. Cannarozzi, J. Alberto Cruz, Liliana M. Dávalos, Noé U. de la Sancha, Prasiddhi Gyawali, Maggie M. Hantak, Samantha Hopkins, Brooks Kohli, Jessica N. King, Michelle S. Koo, A. Michelle Lawing, Helena Machado, Samantha M. McCrane, Bryan McLean, Michèle E. Morgan, Suzanne Pilaar Birch, Denne Reed, Elizabeth J. Reitz, Neeka Sewnath, Nathan S. Upham, Amelia Villaseñor, Laurel Yohe, Edward B. Davis, and Robert P. Guralnick

Supplemental Items

Figure S1. Relationship between average body mass (g) difference and sample size, related to Results, *Case studies*. This is to test if whether having more samples drove the differences seen between average body mass records in FuTRES and PanTHERIA. We did not find a significant relationship (slope = -0.29, R^2 = 0, p-value = 0.857, df = 771). See Results, *Case studies*.

Figure S2. Relationship between average body mass (g) and body mass difference (∆ mass) between FuTRES and PanTHERIA, related to Results, *Case studies*. This is to test whether larger-bodied species, with a wider body mass range, would show a greater difference between the body mass estimates. There is no relationship (slope = 0.453, R^2 = 0.472, p-value <0.001, df = 771). See Results, *Case studies*.

Table S2. Results from sensitivity analyses, related to Figure 3 and Results, *Case studies*. We tested for the effect of sample size (N) and average body mass of a species on the difference in body mass between the FuTRES datastore and PanTHERIA. We present the intercept, standard errors (*se*), slope, p-value, r-squared value (R^2), and degrees of freedom (df). We find that sample size does not drive the difference in mass that we see between FuTRES and PanTHERIA (Figure S1). However, the average size of the species does, where smaller-bodied species show a greater difference in body mass between the two data sources than larger-bodied species (Figure S2). See Figure 3 and Results, *Case studies*.

Table S4. Specimen record information, related to Figure 4, Results, *Case studies*, and STAR Methods. Metadata for modern *Odocoileus virginianus* specimens and measurements used to create the allometric relationship between astragalus lateral length and body mass See Figure 4, Results, *Case studies*, and STAR Methods

Data S1. Minimum required terms for contributing data, related to Figure 1 and STAR Methods. These metadata attributes help make data interoperable across disciplines and are required by the template for upload into GEOME. See Figure 1 and STAR Methods.

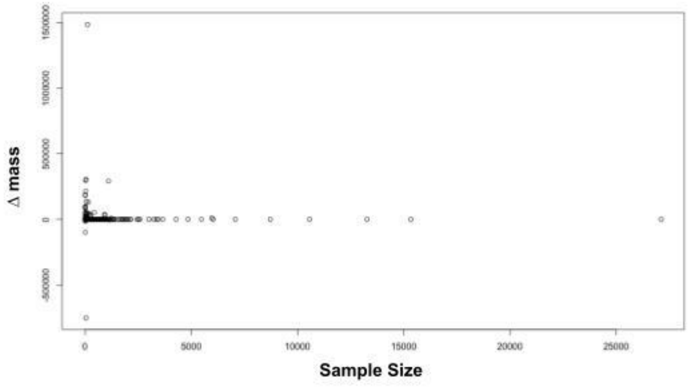
Δ mass
1500000
1000000
500000
0
-500000
0
5000
10000
15000
20000
25000
Sample Size

Figure S1.

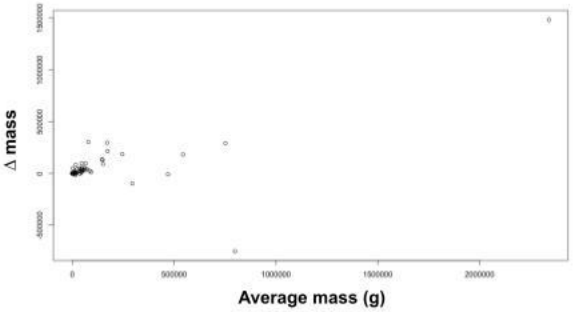
Δ mass
-500000
0
500000
1000000
1500000
0
500000
1000000
1500000
2000000
Average mass (g)

Figure S2.

Table S2. Results from sensitivity analyses, related to Figure 3. We tested for the effect of sample size (N) and average body mass of a species on the difference in body mass between the FuTRES datastore and PanTHERIA. We present the intercept, standard errors (*se*), slope, p-value, r-squared value (R^2), and degrees of freedom (df). We find that sample size does not drive the difference in mass that we see between FuTRES and PanTHERIA (Figure S1). However, the average size of the species does, where smaller-bodied species show a greater difference in body mass between the two data sources than larger-bodied species (Figure S2).

Formula	Intercept (*se*)	Slope (*se*)	p-value	R^2	df
$\Delta\ body\ mass \sim N$	4564.78 (2396.54)	-0.29 (1.61)	0.857	0.00	771
$\Delta\ body\ mass \sim average\ body\ mass$	-104.76 (1700.53)	0.45 (0.017)	<0.001	0.47	771

Table S4. Specimen record information, related to Figure 4. Metadata for modern *Odocoileus virginianus* specimens and measurements used to create the allometric relationship between astragalus lateral length and body mass.

Specimen ID	Sex	Country	County	Locality	Astragalus lateral length (mm)	Body mass (g)
EAP Z2328		USA	Levy	Gad's Bay	37.4	42000.0
EAP Z2773	F	USA	Clay	Camp Blanding	37.7	39500.0
EAP Z2850	F	USA	Clay	Camp Blanding	38.0	45000.0
EAP Z3053	M	USA	Alachua	Lochloosa Wildlife Management	35.1	32100.0
EAP Z3590	M	USA	Marion	Oklawaha River	36.1	41000.0
EAP Z3711	F	USA	Bradford	Camp Blanding	37.6	41500.0
EAP Z4567	F	USA	Liberty	St. Catherines Island	31.5	41500.0
EAP Z4569	F	USA	Collier	Big Cypress	34.5	38590.0
EAP Z4570	F	USA	Collier	Big Cypress	36.5	27240.0
EAP Z4571	M	USA	Collier	Big Cypress	40.2	59928.0
EAP Z4572	F	USA	Collier	Big Cypress	33.1	36320.0
EAP Z4573	F	USA	Collier	Big Cypress	37.8	46308.0
EAP Z4574	F	USA	Collier	Big Cypress	35.0	44038.0
EAP Z4576	F	USA	Collier	Big Cypress	33.3	32688.0
EAP Z4577	M	USA	Collier	Big Cypress	36.9	27687.9
EAP Z4578	M	USA	Collier	Big Cypress	35.4	30844.1
EAP Z4579	M	USA	Collier	Big Cypress	34.8	33142.0
EAP Z4580	F	USA	Collier	Big Cypress	34.1	38590.0
EAP Z4581	F	USA	Collier	Big Cypress	32.7	32688.0
EAP Z4700	F	USA	Collier	Big Cypress	33.4	29510.0
EAP Z4701	F	USA	Collier	Big Cypress	32.3	25424.0
EAP Z4702	F	USA	Collier	Big Cypress	33.3	37228.0
EAP Z4703	F	USA	Collier	Big Cypress	36.1	41768.0
EAP Z4704	F	USA	Collier	Big Cypress	35.9	31780.0
EAP Z4705	F	USA	Collier	Big Cypress	33.7	38590.0
EAP Z4707	F	USA	Collier	Big Cypress	36.4	36320.0
EAP Z4708	F	USA	Collier	Big Cypress	31.5	21792.0
EAP Z4709	F	USA	Collier	Big Cypress	34.9	37228.0
EAP Z4710	M	USA	Collier	Big Cypress	35.2	34504.0
EAP Z4711	M	USA	Collier	Big Cypress	36.7	39952.0

Data S1. Minimum required terms for contributing data. **Related to Figure 1 and STAR Methods.** These metadata attributes help make data interoperable across disciplines and are required by the template for upload into GEOME.

FuTRES Metadata Terms:
individualID: unique identifier for the specimen or collection of elements suspected to be from the same organism.
materialSampleID: unique identifier for the specimen being measured.
diagnosticID: unique identifier for the measurement of an element.
eventide: unique identifier occurrence of a measurement.
materialSampleType: a controlled vocabulary describing the completeness of the specimen.
yearCollected: year in YYYY format derived from verbatimEventDate.

Darwin Core (dwc) terms:
institutionCode
institutionID
collectionCode
catalogNumbner
scientificName
basisOfRecord
lifeStage
measurementType
measurementValue
measurementUnit
measurementMethod
measurementRemarks
measurementDeterminedDate
measuremenetAccuracy
verbatimEventDate
samplingProtocol
locality
country
references [from DCMI Metadata Terms (dc)]
